# Acetate attenuates inflammasome activation through GPR43-mediated Ca^2+^-dependent NLRP3 ubiquitination

**DOI:** 10.1038/s12276-019-0276-5

**Published:** 2019-07-23

**Authors:** Mengda Xu, Zhengyu Jiang, Changli Wang, Na Li, Lulong Bo, Yanping Zha, Jinjun Bian, Yan Zhang, Xiaoming Deng

**Affiliations:** 1 Faculty of Anesthesiology, Changhai Hospital, Second Military Medical University, 200433 Shanghai, China; 2 Department of Anesthesiology, Wuhan General Hospital, PLA, 430070 Wuhan, Hubei Province China

**Keywords:** Acute inflammation, Phosphoinositol signalling, Inflammasome

## Abstract

Acetate has been indicated to be elevated and to regulate inflammation in inflammatory and metabolic diseases. The inflammasome serves as a key component of immune homeostasis, and its dysregulation can lead to various inflammatory disorders. However, little is known about the effects of acetate on inflammasome activation and the underlying mechanism. Here, we demonstrate that acetate attenuates inflammasome activation via GPR43 in a Ca^2+^-dependent manner. Through binding to GPR43, acetate activates the G_q/11_ subunit and subsequent phospholipase C-IP_3_ signaling to decrease Ca^2+^ mobilization. In addition, acetate activates soluble adenylyl cyclase (sAC), promotes NLRP3 inflammasome ubiquitination by PKA, and ultimately induces NLRP3 degradation through autophagy. In vivo, acetate protects mice from NLRP3 inflammasome-dependent peritonitis and LPS-induced endotoxemia. Collectively, our research demonstrates that acetate regulates the NLRP3 inflammasome via GPR43 and Ca^2+^-dependent mechanisms, which reveals the mechanism of metabolite-mediated NLRP3 inflammasome attenuation and highlights acetate as a possible therapeutic strategy for NLRP3 inflammasome-related diseases.

## Inflammatory diseases: Putting on the brakes

Acetate, a short-chain fatty acid, reduces activation of the NLRP3 inflammasome, a multiprotein complex that mediates inflammation. Acetate is produced by gut bacteria and can stimulate the synthesis of fat. High levels of acetate have been associated with diabetes, heart failure and tumor growth, but its effects on the immune system are largely unknown. A study led by Xiaoming Deng and Yan Zhang at the Second Military Medical University in Shanghai, China, shows that acetate has protective effects in mouse models of peritoneal and systemic inflammation and does not cause significant toxicity. Acetate suppresses inflammation by triggering a signaling pathway that reduces the activity of the NLRP3 inflammasome and leads to its degradation. These findings highlight a potential new approach for treating inflammatory diseases.

## Introduction

The inflammasome is a series of multiprotein complexes that serve as a platform to promote interleukin (IL)-1β secretion and pyroptosis^1^. The NACHT, LRR, and PYD domain-containing protein 3 (NLRP3) inflammasome comprises the NOD-like receptor, the adaptor protein ASC and caspase-1 and is considered to play a crucial role in the immune response to pathogens^2^. However, dysregulation of NLRP3 inflammasome activation leads to excessive inflammation, which is linked to various inflammatory disorders, including type 2 diabetes, atherosclerosis, gout, and Alzheimer’s disease^3–5^. Therefore, the regulation of NLRP3 inflammasome activation has emerged as a therapeutic target for inflammasome-related illnesses.

Recently, several metabolites, such as short-chain fatty acids, dopamine and bile acid, have been reported to be involved in NLRP3 inflammasome regulation^5–7^. For example, by binding to transmembrane G-protein-coupled receptor-5 (TGR5) or dopamine receptor D1 (DRD1), metabolites can induce an increase in cyclic adenosine monophosphate (cAMP) and subsequently activate protein kinase A (PKA) and promote NLRP3 ubiquitination and degradation through autophagy or the proteasome^5,6^. cAMP generated by transmembrane or soluble adenylyl cyclase (tmAC or sAC, respectively) is considered to function as a brake for inflammasome activation^5,6,8^. However, it remains unknown whether other metabolites function in a similar manner.

Acetate is a short-chain fatty acid produced by the gut microbiota, and its effects^9–12^, when elevated in specific conditions, are extensive and include modulation of tumor growth and regulation of the endocrine and cardiovascular systems. Gao et al.^9^ regard acetate to be an epigenetic modulator that promotes lipid synthesis in cancer cells. Another study showed that increased concentrations of acetate promoted metabolic syndrome in a parasympathetic signaling-dependent manner^10^. In most cases, the biological effect of acetate depends on its receptor^13^. G-protein-coupled receptor 43 (GPR43) is widely expressed on the surface of innate immune cells, including macrophages, and engages in immune activity. For example, in polymorphonuclear cells, GPR43 functions as a regulator that modulates the activity of neutrophils^14^. GPR43 is a natural receptor for acetate^15^, but most studies have focused on acetate in the context of the gut microbiota and the regulation of inflammatory homeostasis^13,16^. Despite the emerging importance of acetate in the regulation of inflammation, the specific function of acetate in inflammasome regulation and the underlying mechanism remain largely unknown.

Our investigation demonstrates that acetate regulates the NLRP3 inflammasome via GPR43-sAC-PKA signaling and attenuates its activation through ubiquitination and autophagy. Furthermore, acetate also shows anti-inflammatory effects on several peritonitis and endotoxemia models in vivo, indicating that acetate might represent an attractive strategy for treating inflammasome-related diseases.

## Materials and methods

### Animal model and experiments

Six- to 8-week-old male C57BL/6J mice were purchased from the Shanghai Laboratory Animal Center (SLAC) (China). All mice were housed at a temperature of 18–22 °C with a relative humidity of 50–60% and a 12-h light–dark cycle, with free access to water and food. Animal experiments were approved by the Scientific Investigation Board of Second Military Medical University. For the mouse peritonitis model, each mouse was pretreated with 40 µl of 2.5 M acetate (acetate solution, pH 5.2, Sigma-Aldrich, Lot. 3863) diluted in 400 µl of PBS. Thirty minutes later, the mice were injected with alum (2 mg/mouse; Thermo Scientific, China) or monosodium urate (MSU) (2 mg/mouse; Sigma, China) diluted in 50 µl of PBS or with 10 mg/kg lipopolysaccharide (LPS) (*Escherichia coli* O111:B4, Sigma, China) diluted in 300 µl of PBS; 6 h after the MSU or alum injection, peritoneal lavage was performed with 5 ml of normal saline (NS), and the lavage fluid was centrifuged.

### Cell preparation and stimulation

For peritoneal macrophage (PM) generation, each mouse was injected with thioglycollate (BD, USA). Three days after the injection, peritoneal lavage was performed with 5 ml of NS. The cells were resuspended at 2–4 × 10^6^ cells/ml and cultured in RPMI 1640 culture medium supplemented with 10% fetal bovine serum (FBS). For bone marrow-derived macrophage (BMDM) generation, mouse femoral tissue was isolated, and the bone marrow was flushed with 3 ml of NS. After red blood cell lysis, bone marrow cells were resuspended at 2–4 × 10^6^ cells/ml in Dulbecco’s modified Eagle’s medium (DMEM) supplemented with 10% FBS and 30 ng/ml GM-CSF (PeproTech, USA). The culture medium was changed every 2 days, and after 5–6 days of culture, the cells were subjected to further experiments. To activate the inflammasome in PMs or BMDMs, the cells were first primed with LPS (100 ng/ml) for 3 h, and ATP (5 mM), nigericin (20 µM), muramyl dipeptide (MDP) (200 ng/ml), flagellin (10 µM) or poly(dA:dT) (1 µg/ml) was added for 30 min. The supernatant was subjected to further analysis. Inhibitors (KH7, H89 and bafilomycin A1 from Cayman Chemical, USA; MG-132 from Selleck, USA; and 3-MA from Merck, Germany) or agonists (GPR43 agonist, Millipore, USA) were added to the culture medium 30 min before acetate pretreatment and after LPS priming.

### Cytokine ELISA, lactate dehydrogenase (LDH) assay, and cAMP measurement

IL-1β, IL-6, and tumor necrosis factor (TNF)-α were analyzed using an ELISA kit purchased from Invitrogen (USA), and IL-18 was analyzed using a kit from R&D Systems (USA). LDH release assays were performed using a Cytotoxicity Detection Kit purchased from Roche Life Science (USA). cAMP measurement was performed by using a cAMP Parameter Assay Kit (R&D Systems, USA).

### Immunoblotting and immunoprecipitation

Cells were lysed in radioimmunoprecipitation assay (RIPA) buffer (Beyotime, China), and protein concentrations were determined using a BCA assay (Thermo, China). The total protein samples (approximately 20 μg) were separated by sodium dodecyl sulfate polyacrylamide gel electrophoresis, transferred to polyvinylidene fluoride (PVDF) membranes (Merck, Germany), and blocked with 5% nonfat dry milk in phosphate-buffered saline with Tween (PBST), pH 7.5. The membranes were immunoblotted with primary antibodies for 4 h or overnight at 4 °C and then incubated with horseradish peroxidase (HRP)-conjugated secondary antibodies. The anti-ASC, anti-NLRP3, anti-p62, anti-LC3B, anti-β-actin, anti-K63, anti-K48, anti-pro-IL-1β, anti-phospho-IKKβ, anti-IKKβ, anti-phospho-p65, and anti-p65 antibodies were purchased from Cell Signaling Technology (MA, USA). Other antibodies used in the present study were as follows: anti-pro-caspase-1 (Adipogen, USA), anti-GPR43 (Absin, China), anti-GPR41 (Thermo Fisher, USA), anti-MARCH7 (Bioss, China), and anti-sAC (FabGennix, USA). The HRP-conjugated secondary antibodies were all purchased from Cell Signaling Technology (USA). Protein bands were detected with an enhanced chemiluminescence kit (Pierce, USA).

For the immunoprecipitation assay, briefly, the cells were collected and lysed using a mortar and pestle in a buffer containing 20 mM PIPES, pH 6.8, 1% Triton X-100, 150 mM NaCl, 150 mM sucrose, 0.2% sodium deoxycholate, 500 μM EDTA and protease inhibitors for 5 min on ice. After centrifugation, the supernatants were diluted to 2 μg/ml in dilution buffer containing 20 mM PIPES, pH 6.8, 1% Triton X-100, 150 mM NaCl, 150 mM sucrose, 2.5 mM MgCl_2_ and 2.5 mM MnCl_2_. Primary antibody-conjugated protein A beads were incubated with the lysates for 2 h at 4 °C before washing with dilution buffer. The subsequent immunoblot analysis was conducted using the abovementioned methods. The chemicals were purchased from Sigma.

### Flow cytometry

Peritoneal lavage cells from individual mice were prepared in PBS and stained with antibodies at 4 °C for 15 min. After washing three times, the cells were resuspended in washing buffer and analyzed. Fluorescence data for 10^5^ events from each sample were acquired on a FACS LSR II (BD Bioscience, USA) and analyzed using FlowJo software (Tomy Digital Biology Co., Ltd., Japan). The antibodies used in these experiments (neutrophils: anti-mouse-CD11b and anti-mouse-GR-1; macrophages: anti-mouse-F4/80) were purchased from eBioscience or Invitrogen (USA).

### Short-interfering RNA (siRNA) interference

Mouse PMs or BMDMs were cultured in half of the final total culture volume in FBS-free RPMI 1640 or DMEM and transfected with 3 ng/ml siRNA (si-GPR43, si-GPR41, si-MARCH7, si-sAC1, si-sAC2, or si-Gq) or control siRNA (GenePharma, China) by INTERFEREin (Invitrogen, USA) for 6 h according to the manufacturer’s instructions. Six hours later, the other half of the complete culture medium was added, and the cells were cultured for a total of 48 h. After 48 h of interference, the cells were subjected to further stimulation or experiments. The sequences of each siRNA were as follows: si-GPR43: GCUGGUACCUACCAAAGAUdTdT; si-GPR41: GCUUCUUUCUUGGCAAUUAdTdT; si-MARCH7: GGACUUAUGUAGAAUUUGUdTdT; si-sAC1: AAUGUAUGGGCUUCAUGGAdTdT; si-sAC2: TCGGAGCATGATTGAAATCGA; and si-Gq: AAAUGACACUUUGUAAGUCAAAGGG.

### Cytosolic calcium analysis

Macrophages were primed with 100 ng/ml LPS (*E. coli* O111:B4, Sigma, China) for 3 h. After two washes, the cells were incubated in HBSS containing 2 µM Fluo 4-AM (Life Technologies, USA) for 30 min in the dark. The cells were then washed, treated with or without acetate (20 mM) for 30 min, treated with 2-APB (50 µM) (Sigma-Aldrich, USA) for 15 min, resuspended in HBSS and added to black 96-well plates (Corning Costar, USA). After 10 min of incubation, the cells were stimulated with nigericin (20 µM) (Sigma-Aldrich, USA). A DMI6000 inverted fluorescence microscope (Leica, Germany) with an X-Cite® 200DC fluorescent light source (Lumen Dynamic, Canada) was used for fluorescence analysis. Fluorescence emission at 520 nm and excitation at 485 nm were used. ΔFluorescence was calculated as *F*_max_ (fluorescence of the calcium-saturated indicator) minus F_0_ (fluorescence in nonstimulated cells in HBSS).

### Statistical analysis

Data were expressed as the mean ± SEM. Student’s *t*-test was conducted for comparisons between two groups, and one-way ANOVA was performed for comparisons among several groups. A *P*-value < 0.05 was considered to be statistically significant.

## Results

### Acetate attenuates NLRP3-mediated inflammasome activation in vitro and in vivo

**Fig. 1:**
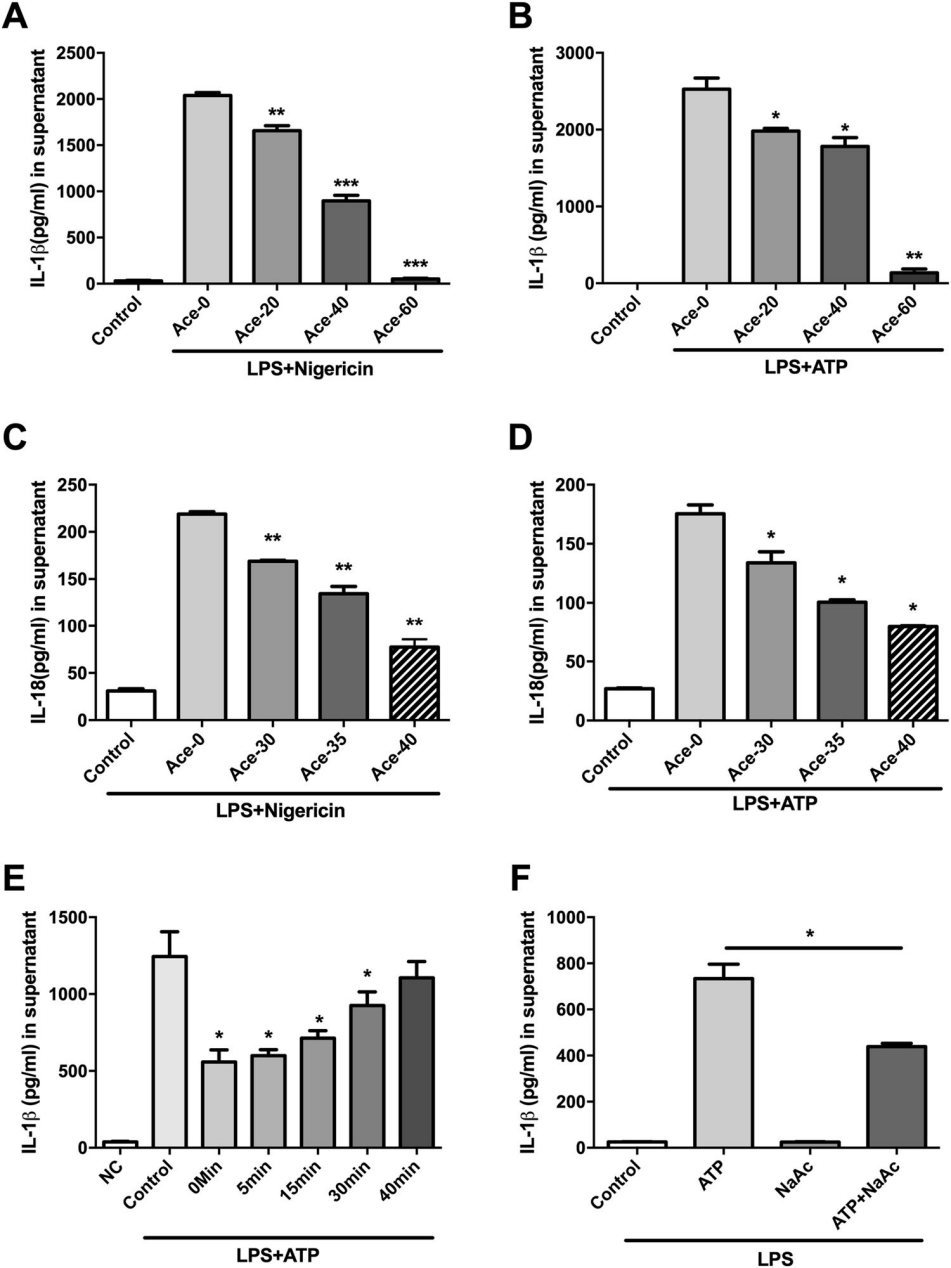
Acetate attenuates inflammasome activation in vitro. **a**–**d** ELISA of IL-1β (**a**, **b**) and IL-18 (**c**, **d**) in BMDMs treated with LPS + nigericin or LPS + ATP at different doses (20, 30, 35, 40, and 60 mM) (*n* = 3); **e** ELISA of IL-1β after sodium acetate treatment (*n* = 3); **f** ELISA of IL-1β at different time points after acetate treatment in LPS-primed ATP-treated BMDMs (*n* = 3); *n* = biological replicates; Data are presented as the mean ± SEM. *P < 0.05, **P < 0.01, and ***P < 0.001, Student’s *t-*test compared with the control group

To explore the anti-inflammatory effect of acetate on NLRP3 inflammasome activation, LPS-primed BMDMs were treated with acetate before nigericin stimulation. The concentrations of IL-1β and IL-18 in the supernatant were tested. We found that acetate suppressed the ATP- or nigericin-induced production of IL-1β and IL-18 in a dose-dependent manner (Fig. 1a–d). In addition, the attenuation of cytokine production could also be observed when acetate was added after LPS and nigericin activation (Fig. 1e). The acetate treatment had little effect on LDH production (Supplemental Fig. 1), which suggests that the inhibitory effect was not due to damage to the cells.

**Fig. 2:**
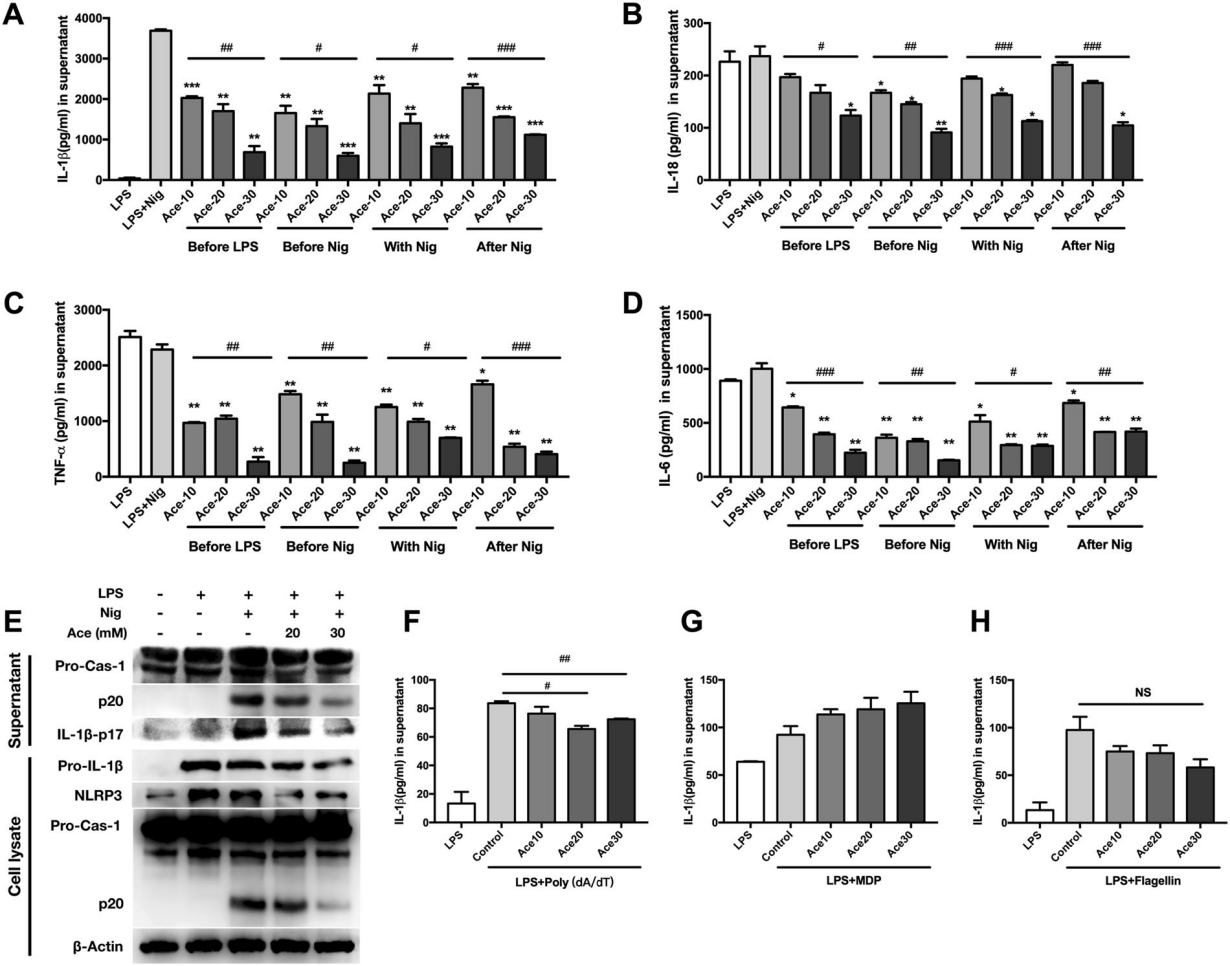
Acetate suppresses NLRP3 inflammasome activation at different time points in vitro. **a**–**d** ELISA of IL-1β (**a**), IL-18 (**b**), TNF-α (**c**), and IL-6 (**d**) production after acetate treatment in BMDMs. Acetate (10, 20, and 30 mM) was administered 30 min before LPS priming, 30 min before nigericin stimulation, simultaneously with nigericin or 30 min after nigericin stimulation. All treatments showed suppressive effects in a dose-dependent manner (*n* = 3); *P < 0.05, **P < 0.01, and ***P < 0.001, Student’s *t-*test compared with the LPS + Nig group; ^#^*P* < 0.05, ^##^*P* < 0.01, and ^###^*P* < 0.001, one-way ANOVA for comparison. **e** Western blot of pro-caspase-1, caspase-1-p20, and IL-1β-p17 in supernatant and NLRP3, pro-caspase-1, caspase-1-p20 and β-actin in cell lysate after indicated treatment; **f**–**h** ELISA of IL-1β after treatment with poly(dA/dT), MDP and flagellin (*n* = 3). *n* = biological replicates. Data are presented as the mean ± SEM

We further performed comprehensive experiments investigating the role of acetate in suppressing inflammation and the inflammasome. We found that acetate suppressed IL-1β and IL-18 production in response to pretreatment, simultaneous treatment or post treatment with LPS and nigericin (Fig. 2a, b). Moreover, acetate decreased TNF-α and IL-6 production by LPS-primed BMDMs (Fig. 2c, d) and partly attenuated NF-κB signaling activation accordingly (Supplemental Fig. 2). In the further analysis of NLRP3 inflammasome activation in response to acetate pretreatment, we found that acetate could decrease the level of cleaved caspase-1 (p20) in the supernatant and cell lysate of BMDMs and NLRP3 in cell lysates (Fig. 2e), indicating the inhibitory effect of acetate on NLRP3 inflammasome activation.

We also determined that acetic acid, rather than hydrogen ions, exerts these inhibitory effects because sodium acetate had an inhibitory effect similar to that of acetate (Fig. 1f). These results indicated that the suppression mediated by acetate was independent of the treatment period, which suggests that acetate exerted its inhibitory effect on the inflammasome during the activation stage.

Different inflammasomes respond to different signals and trigger different responses^17^. To determine whether acetate impacts other types of inflammasomes, we tested the impact of acetate on the Absent in melanoma 2 (AIM2), NLR family CARD domain-containing protein 4 (NLRC4) and NACHT, LRR and PYD domain-containing protein 3 (NALP3) inflammasomes after activation by poly(dA:dT), flagellin or MDP. Acetate showed no detectable inhibitory effect (NLRC4 and NALP3) or relatively limited attenuation (AIM2) on these inflammasomes (Fig. 2f, g). Taken together, our results show that acetate suppresses IL-1β production mediated by the NLRP3 inflammasome.

**Fig. 3:**
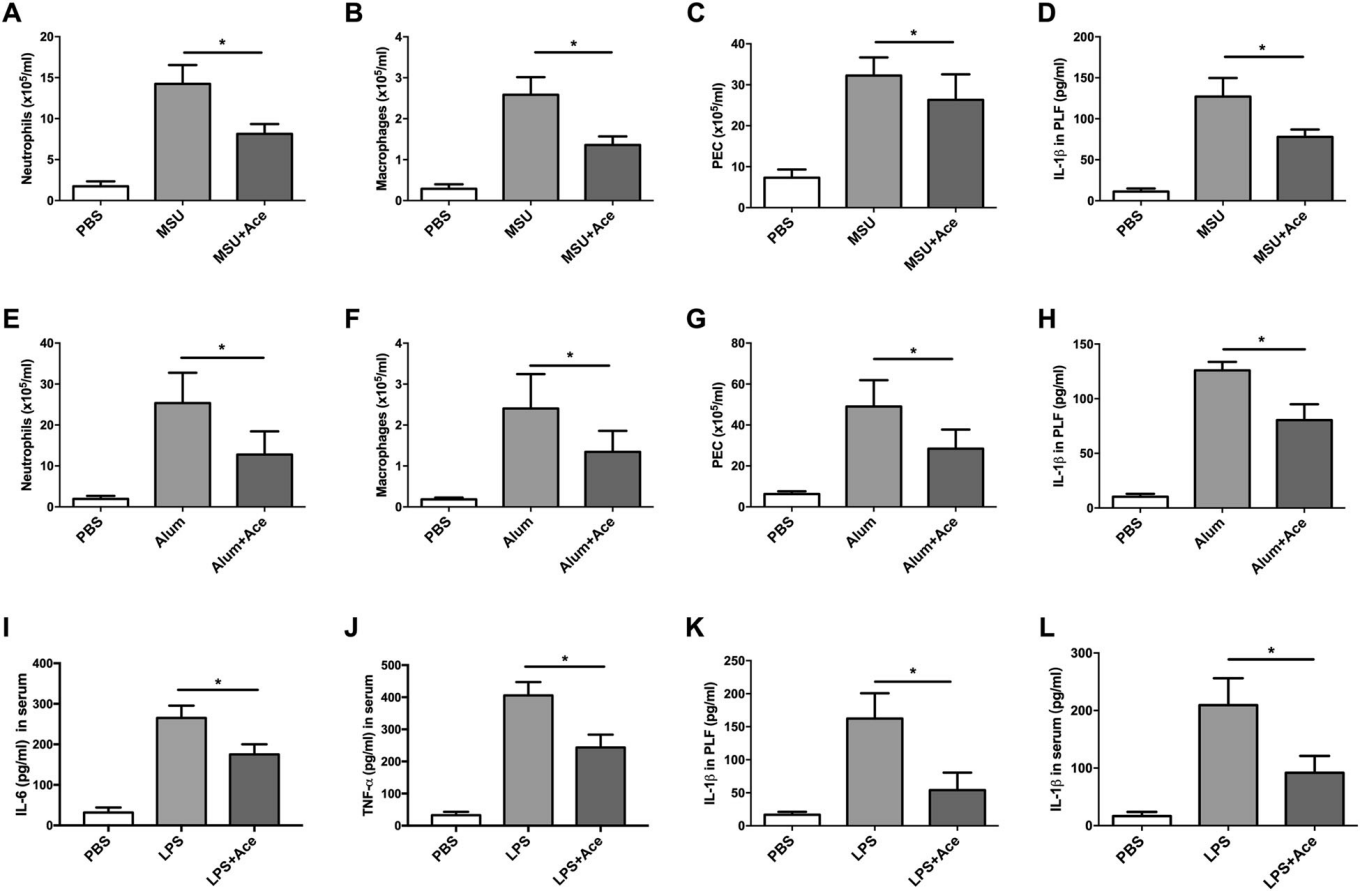
Acetate suppresses inflammasome-mediated peritoneal inflammation in vivo. **a**–**h** Acetate (20 mM) suppressed MSU- (**a**–**d**) and alum-(**e**–**g**)-induced peritoneal inflammation. Flow cytometry revealed that neutrophils (CD11b^+^ GR-1^+^) (**a**, **e**), macrophages (F4/80^+^) (**b**, **f**), peritoneal exudate cells (PECs) (**c**, **g)** and IL-1β (ELISA) in the peritoneal lavage fluid (PLF) (**d**, **h**) were decreased in the acetate pretreatment group; **i**–**l** acetate suppressed LPS-induced peritonitis (*n* = 3). Serum levels of IL-6 (**i**), TNF-α (**j**), IL-1β (**l**), and IL-1β in PLF (**k**), as measured by ELISA, decreased in the acetate treatment group (*n* = 3); *P < 0.05, Student’s *t-*test compared with the model group (MSU, Alum or LPS). *n* = biological replicates. Data are presented as the mean ± SEM

We further evaluated the anti-inflammatory effect of acetate in vivo. To adapt MSU- and alum-induced peritoneal inflammation^3,18^, which have been proven to be NLRP3 inflammasome-dependent models, and LPS-induced peritonitis, we pretreated mice with acetate intraperitoneally to investigate its anti-inflammatory effects. Peritoneal lavage fluid (PLF), peritoneal exudate cells (PECs) and serum were harvested for further assessments. As shown in Fig. 3 and Supplemental Fig. 3, compared with those of the control mice, IL-1β levels and the numbers of PECs, neutrophils and macrophages were all decreased in acetate-treated mice during MSU- or alum-induced peritonitis (Fig. 3a–h). Moreover, acetate also suppressed the secretion of IL-6 and TNF-α in serum and IL-1β in serum and PLF during LPS-induced peritonitis (Fig. 3i–l). Collectively, these results demonstrated that acetate could suppress inflammation in vivo and attenuate NLRP3-mediated inflammasome activation in vitro and in vivo.

### Acetate-induced attenuation of the NLRP3 inflammasome is mediated by GPR43-G_q/11_-Ca^2+^

**Fig. 4:**
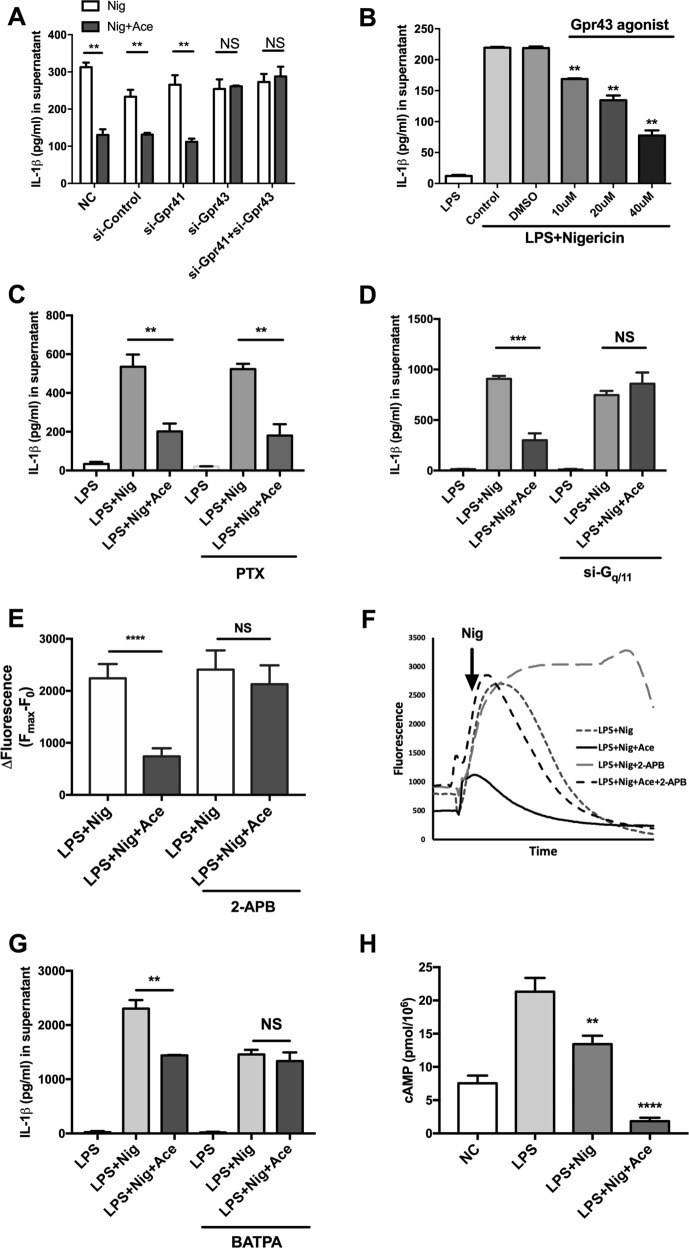
Acetate-induced suppression of the NLRP3 inflammasome is mediated by GPR43-G_q/11_-Ca^2+^. **a**, **b** Acetate-induced suppression of the NLRP3 inflammasome is dependent on GPR43. ELISA of siRNA interference for GPR43 but not GPR41 (**a**) in LPS-primed BMDMs pretreated with acetate for 30 min (*n* = 6). ELISA showing the effect of a GPR43 agonist on IL-1β production (**b**) (*n* = 3). **c**, **d** Acetate-induced suppression of the NLRP3 inflammasome requires the G_q/11_ subunit rather than the G_I/O_ subunit. ELISA of IL-1β in G_I/O_ subunit inhibitor-treated (**c**) and G_q/11_ subunit siRNA knockdown (**d**) LPS-primed BMDMs (*n* = 6). **e**, **f** Fluorescence change of *F*_max_ and *F*_0_ and representative curve of calcium changes in PMs with or without treatment with 2-APB (*n* = 4). **g** Chelation of Ca^2+^ (BAPTA) reversed the suppressive effects of acetate on IL-1β production (*n* = 3). **h** cAMP levels measured by ELISA were decreased by acetate in LPS + Nig-treated BMDMs (*n* = 3). *n* = biological replicates. Data are presented as the mean ± SEM. *P < 0.05, **P < 0.01, ***P < 0.001, and ****P < 0.0001, Student’s *t-*test compared with the control group

We next analyzed the possible mechanism by which acetate mediates inflammasome suppression. Acetate has been proven to be capable of activating short-chain fatty acid receptors, including GPR41 and GRP43^15^. Previous studies have reported the anti-inflammatory effects of GPR43/41 in several biological processes, and among short-chain fatty acids, acetate is one of the strongest agonists of GPR43^15^. Therefore, we hypothesized that acetate exerts its inhibitory effect by activating a short-chain fatty acid receptor. To validate this hypothesis, we knocked down Gpr43 or Gpr41 in BMDMs (Supplemental Fig. 4A, B) and treated the cells with acetate before nigericin stimulation. The results showed that the knockdown of Gpr43 reversed the effect of acetate on the inflammasome, while the knockdown of GPR41 had no effect (Fig. 4a). We further used an agonist of GPR43 in BMDMs to validate the suppression of IL-1β and IL-18 in the supernatant. We found that the GPR43 agonist had an inhibitory effect similar to that of acetate (Fig. 4b), suggesting that acetate inhibited the inflammasome via GPR43 signaling.

Then, we asked why GPR43, but not GPR41, was required because acetate has an affinity for both receptors. Previous studies discovered that GPR41 couples to the G_I/O_ subunit while GPR43 couples to either the G_I/O_ or G_q/11_ subunit^16^. We hypothesized that different G-protein subunits may enable GPR43 to perform distinct biological functions. Therefore, we used pharmacological and genetic methods to determine which subunit was involved in the inhibitory effect of acetate on the NLRP3 inflammasome. We found that treating BMDMs with pertussis toxin (PTX), a G_I/O_ subunit inhibitor, did not alter the inhibitory effect of acetate, whereas knockdown of the G_q/11_ subunit (Supplemental Fig. 4C) abolished the suppression of IL-1β secretion, suggesting that the G_q/11_ subunit is essential for the effect of acetate (Fig. 4c, d).

Previous research reported that the G_q/11_ subunit could couple with phospholipase C (PLC)^19,20^. This interaction may enable GPR43 to engage phosphatidylinositol signals when bound to acetate and subsequently lead to the hydrolysis of phosphatidylinositol bisphosphate (PIP_2_) to inositol-1,4,5-triphosphate (IP_3_) and diacylglycerol (DAG); these products activate protein kinase C (PKC) and InsP_3_R, increasing the concentration of intracellular calcium and eventually promoting NLRP3 inflammasome activation^8,21^. Thus, we analyzed the changes in Ca^2+^ during acetate treatment. Using the Fluo 4-AM cytosolic calcium indicator, we found that acetate decreased cytosolic calcium levels after stimulation with nigericin, and this attenuation of nigericin-induced Ca^2+^ mobilization by acetate required InP_3_R participation, as the effect was reversed when InP_3_R signaling was inhibited by 2-APB (Fig. 4e, f and Supplemental Videos 1–4).

Next, we determined whether Ca^2+^ is required for these effects. Using BAPTA, a specific chelator of Ca^2+^, we found that the inhibitory effect of acetate on NLRP3 inflammasome activation was abrogated, suggesting that Ca^2+^ is essential for the mechanism by which acetate affects the NLRP3 inflammasome (Fig. 4g). Collectively, these results suggest that acetate prevents NLRP3 inflammasome activation via GPR43-G_q/11_-Ca^2+^ signaling.

### Acetate-mediated attenuation of the NLRP3 inflammasome depends on sAC-PKA signaling

**Fig. 5:**
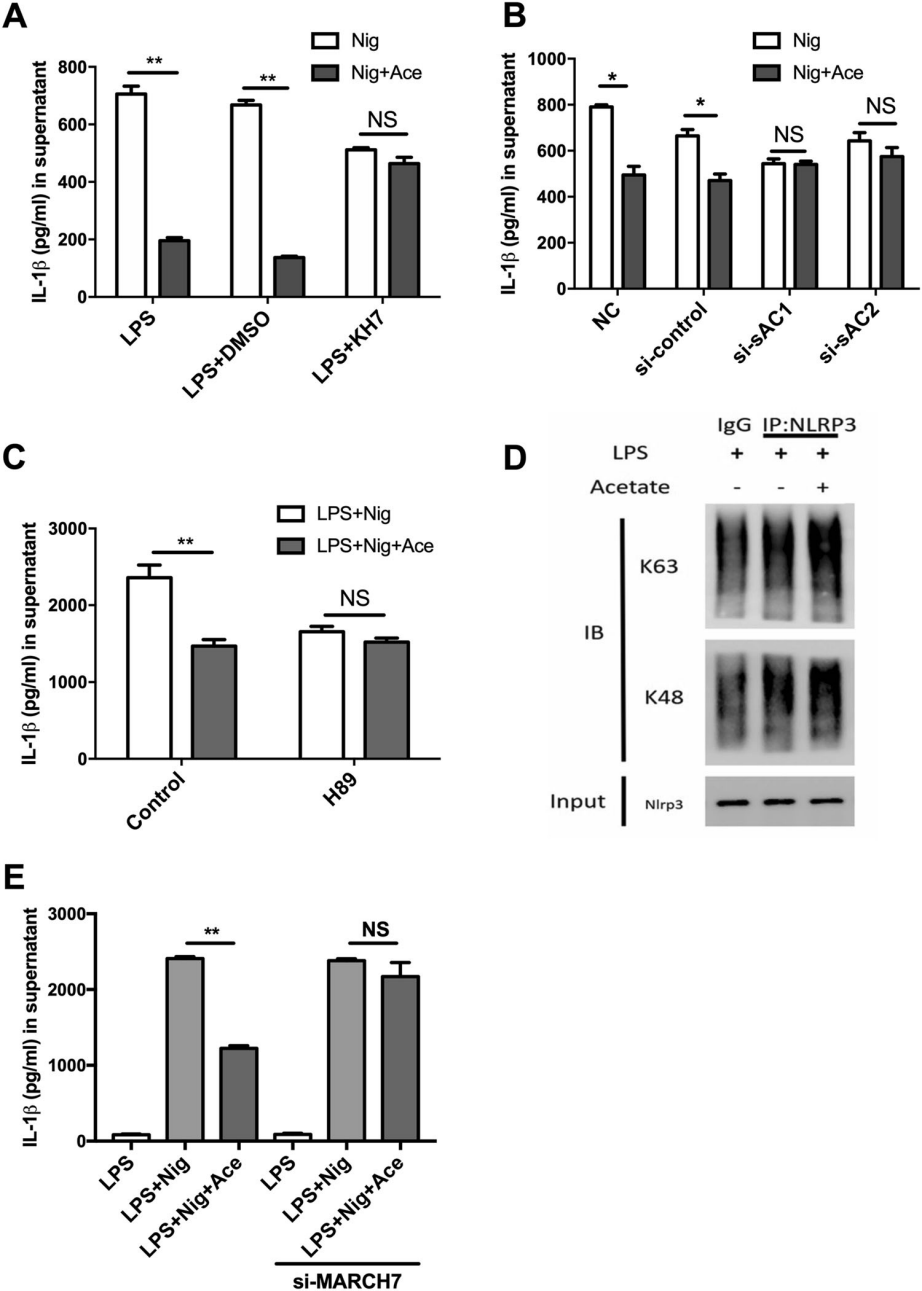
Acetate-induced suppression of the NLRP3 inflammasome occurs via the sAC-PKA axis and promotes polyubiquitination of NLRP3. **a**, **b** ELISA of IL-1β indicated that acetate (20 mM) suppression in BMDMs was dependent on sAC. Inhibition of sAC (KH7) (**a**) or siRNA interference for two types of sAC (**b**) reversed the suppressive effects of acetate (*n* = 6). **c** ELISA of IL-1β indicated that the inhibition of PKA (H89) reversed the suppressive effects of acetate in BMDMs (*n* = 3). **d** Immunoprecipitation of NLRP3 polyubiquitination. The K48 and K63 ubiquitin chains were enhanced after acetate treatment (*n* = 3). **e** ELISA of IL-1β indicated that siRNA interference of MARCH7, an E3 ligase involved in NLRP3 ubiquitination, reversed the suppressive effects of acetate in BMDMs (*n* = 6). *n* = biological replicates. Data are presented as the mean ± SEM. *P < 0.05 and **P < 0.01, Student’s *t-*test compared with the control group

We next investigated the mechanism of GPR43-G_q/11_-Ca^2+^ signaling in the prevention of NLRP3 inflammasome activation. cAMP, which is generated by adenylyl cyclase, is known to serve as a brake for inflammasome activation. However, the total intracellular cAMP level was reduced during acetate treatment (Fig. 4h). Thus, we further investigated the possible underlying mechanism of NLRP3 inflammasome attenuation. Soluble adenylyl cyclase (sAC) is distributed throughout the cell due to its structure, which is distinct from that of tmAC^22–25^, and sAC can be activated by Ca^2+^ to further activate downstream signaling pathways^26,27^. Therefore, we determined whether sAC is essential for NLRP3 inflammasome depression by acetate. First, we found that treatment with KH7, a sAC and tmAC inhibitor^5,6,28^, reversed the inhibitory effect of acetate, whereas treating BMDMs with KH7 alone did not induce IL-1β secretion (Fig. 5a). Second, consistent with previous data, acetate failed to inhibit IL-1β secretion when sAC was knocked down (Supplemental Fig. 4D and Fig. 5b). These results suggest that acetate may exert anti-inflammatory effects through interactions with sAC.

sAC converts ATP into cyclic AMP and consequently activates protein kinase A (PKA). Therefore, we investigated the role of PKA in acetate-induced NLRP3 inflammasome inhibition. H89, a selective inhibitor of PKA, was applied to LPS-primed BMDMs before acetate treatment. The results showed that H89 blocked acetate-induced inflammasome inhibition (Fig. 5c). Collectively, our data demonstrate that the inhibitory effect induced by acetate occurs in a sAC-PKA-dependent manner.

### Acetate induces NLRP3 inflammasome polyubiquitination and autophagy

The relationship between ubiquitination and GPCR signaling has been investigated previously^29^, and growing evidence suggests that ubiquitination links GPCR signaling and inflammasome regulation^5,6,8^. Thus, to determine the role of GPCR signaling-driven ubiquitination in our study, we assessed the ubiquitination level of the NLRP3 inflammasome in BMDMs after treatment with acetate. We found that acetate promoted polyubiquitination of the NLRP3 inflammasome, which contained a mix of K48 and K63 ubiquitin chains (Fig. 5d). In addition, we found that RNA interference of MARCH7 (Supplemental Fig. 4E), a reported E3 ligase that promotes NLRP3 ubiquitination, led to the reversal of the acetate-induced IL-1β reduction (Fig. 5e). These results indicate that NLRP3 inflammasome polyubiquitination could be an essential event for the acetate-mediated suppression of NLRP3 inflammasome activation.

**Fig. 6:**
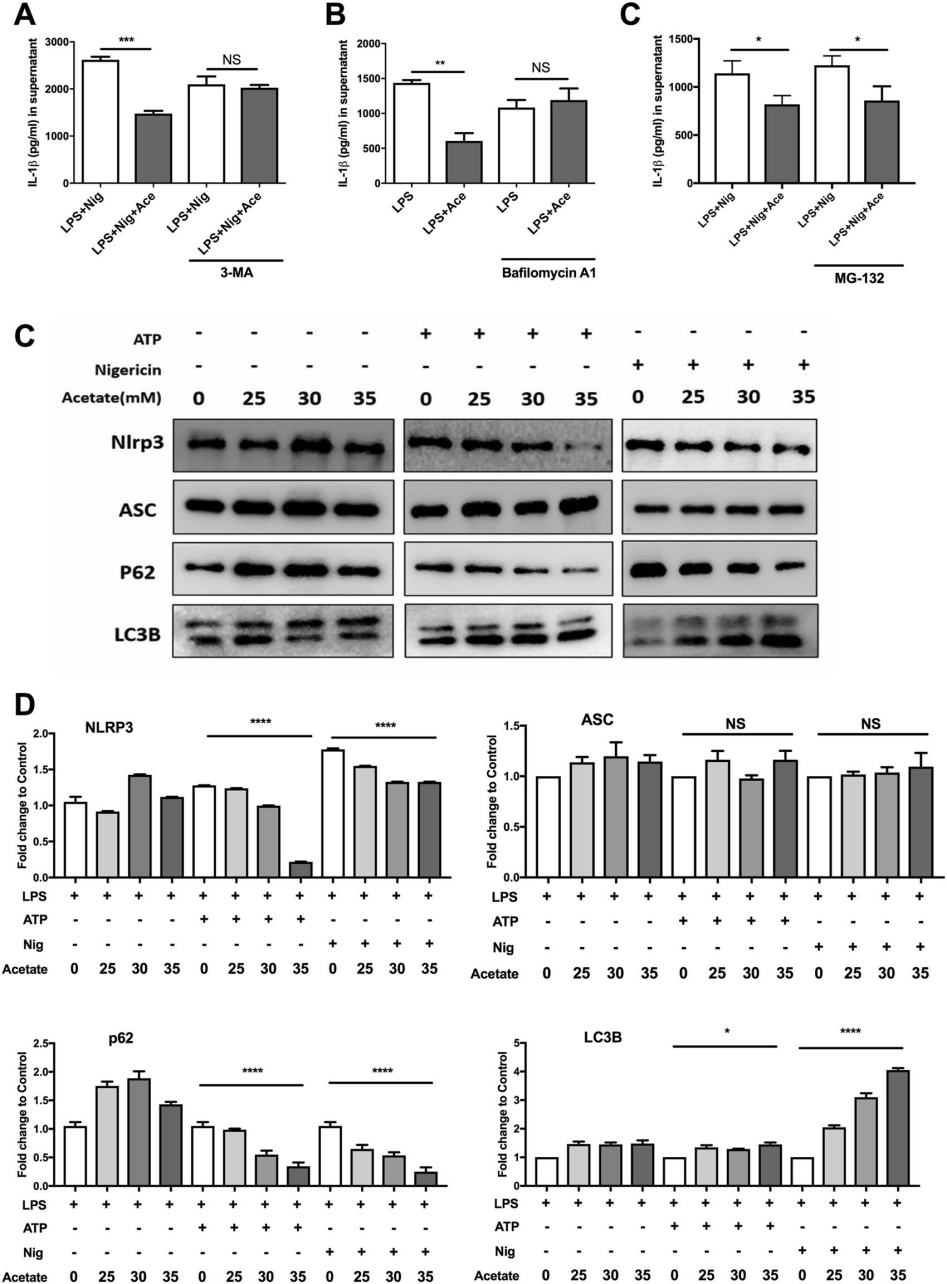
Acetate promotes NLRP3 degradation through autophagy. **a**–**c** ELISA of IL-1β indicated that acetate (25, 30, and 35 mM) mediated suppression of the NLRP3 inflammasome in a manner dependent on autophagy but not the proteasome. Inhibition of autophagy (3-MA and bafilomycin A1) reversed the suppression of IL-1β (**a**, **b**), whereas inhibition of the proteasome induced few alterations (**c**) in BMDMs (*n* = 3). **c**, **d** Western blot of NLRP3, ASC, p62, and LC3B. The degradation of NLRP3 was accompanied by increased NLRP3 expression and decreased p62 expression in BMDMs (*n* = 3). *n* = biological replicates. Data are presented as the mean ± SEM. *P < 0.05, **P < 0.01, ***P < 0.001, and ****P < 0.0001, Student’s *t-*test compared with the control group (**a**–**c**) or one-way ANOVA for comparisons of differences (**d**)

**Fig. 7:**
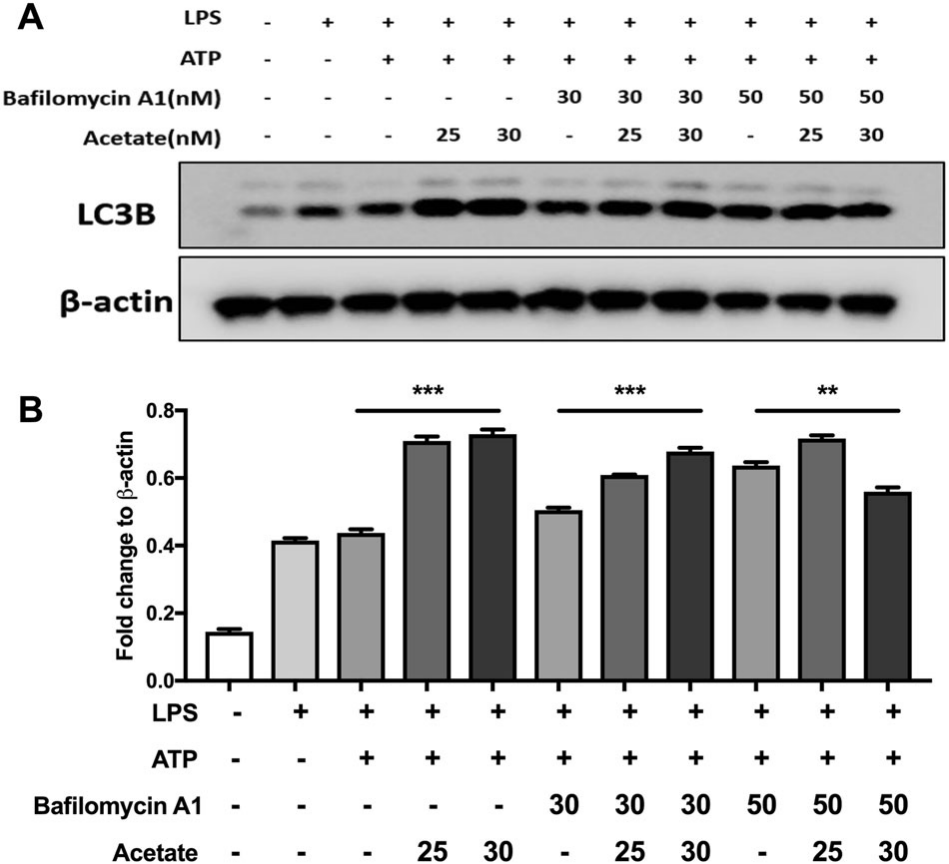
Acetate enhances autophagy in a dose-dependent manner. **a**, **b** Western blot (**a**) and quantification (**b**) of LC3B after treatment with bafilomycin A1 and acetate (mM) (*n* = 3) in BMDMs. *n* = biological replicates. Data are presented as the mean ± SEM. **P < 0.01 and ***P < 0.001, one-way ANOVA for comparisons of differences

We further investigated whether autophagy or the proteasome participated in ubiquitination and protein degradation of the NLRP3 inflammasome. The results showed that both 3-MA and bafilomycin A could abolish the preventive effect of acetate on IL-1β secretion, while MG-132 could not (Fig. 6a–c), suggesting that autophagy, rather than proteasome-mediated proteolysis, was involved in acetate-induced NLRP3 inflammasome inhibition. We further measured NLRP3 and ASC in LPS-primed macrophages treated with acetate. We found that acetate promoted NLRP3 degradation but did not affect ASC, and this alteration was consistent with the changes in LC3B and p62 (Fig. 6c, d). In addition, after treatment with bafilomycin A1, which inhibits autophagy, we found that acetate increased the accumulation of LC3B, which indicates that acetate increased the formation of autophagosomes in a dose-dependent manner (Fig. 7a, b). These results suggest that acetate promotes degradation of the NLRP3 inflammasome by ubiquitination and subsequent autophagy.

## Discussion

Free fatty acids (FFAs), a diverse class of aliphatic hydrocarbon chains with a carboxyl group at one end, have been reported to participate in numerous biological processes, including immune regulation^30^. Different FFAs play distinct roles; for example, palmitate promotes IL-1β and IL-18 secretion in a dose-dependent manner^7^, whereas omega-3 fatty acids can inhibit the NLRP3 inflammasome via GPR120 and GPR40^31^. Very recently, the role of acetate in type 2 diabetes, heart failure and tumors has also been highlighted^7,9,12^. Acetate is present at a relatively low concentration in the blood^32^, but under specific conditions, acetate concentrations can increase to millimolar levels and exert a number of effects^9,10,12^. In vitro studies focusing on the effects of acetate at the cellular and molecular levels have generally applied a 10–20 mM concentration to mimic the effects of acetate in the microenvironment^11,13^. In the present study, we found that the anti-inflammatory effects of acetate were dose-dependent with no significant cytotoxicity, which is consistent with the results of previous research^11^.

Acetate shows a similar affinity for GPR41 and GRP43^15^. The present study showed that the acetate-mediated suppression of NLRP3 inflammasome activation relied on GPR43. We believe this effect may be due to the coupling GPRs to different signaling subunits. GPR43 exhibits dual coupling to the G_I/O_ subunit and the G_q/11_ subunit but not to the G_S_ subunit^4^. Specific activation of GPR43 subsequently influences cAMP and Ca^2+^ and thus activates downstream signaling^4^, and the G_q/11_ subunit is capable of activating PLC and IP_3_ to activate downstream signaling pathways^20,33,34^. Consistent with these conclusions, our results proved that acetate could decrease Ca^2+^ levels after stimulation and that this process required IP_3_R participation, as the blockage of IP_3_R reversed the Ca^2+^ decrease after acetate treatment. Moreover, although acetate decreased Ca^2+^ levels, the signal still required Ca^2+^ participation because the chelation of calcium also abolished the acetate-mediated attenuation of IL-1β. These results indicate that G_q/11_-Ca^2+^ signaling is a possible mechanism for the acetate-mediated attenuation of the NLRP3 inflammasome and highlight the dual role of Ca^2+^ in signal transduction.

However, studies of calcium-sensing receptor (CaSR) found that increased Ca^2+^, accompanied by decreased cAMP, could further promote NLRP3 inflammasome activation^8^, which is inconsistent with the present finding. To address this issue, we focused on the crucial role of cAMP in inflammasome regulation. In recent years, several works found that cAMP could serve as a brake for the NLRP3 inflammasome. Dopamine and bile acid suppress the NLRP3 inflammasome via DR1 signaling or TGR5 signaling^5,6^ and consequently enhance the production of cAMP. The enhanced cAMP can further bind to NLRP3 directly to induce the ubiquitination of NLRP3 through PKA and thus suppress inflammasome activation^5,6^. Thus, the balance of Ca^2+^ and cAMP signaling determines the exact outcome of inflammasome activation^35^. cAMP is generated by two types of adenylyl cyclases that are present in different cellular compartments: the transmembrane adenylyl cyclase (tmAC) within the plasmalemma, and the soluble adenylyl cyclase (sAC) in the cytosol and within distinct organelles^36–39^. Unlike tmAC, sAC can be activated by Ca^2+^, generates cAMP^40^ and may mediate NLRP3 inflammasome attenuation. Indeed, although cAMP concentrations remain relatively low during acetate treatment, we still found an essential role for sAC in the acetate-mediated attenuation of IL-1β production, as both the KH7 inhibitor and siRNA interference of sAC reversed acetate-induced IL-1β attenuation. Hence, the present finding may raise the question of how the cell coordinates the convergence between two different sources of cAMP in regulating the ubiquitination of the NLRP3 inflammasome. Previous studies focused on the regulatory effect of cAMP produced by tmAC on NLRP3 degradation^5,6,41^, but few studies have focused on cAMP synthesized by sAC. In fact, the cAMP signal generated by tmAC is thought to be confined to defined compartments by PDE activity, which makes it difficult for the cAMP signal to be transmitted throughout the cytosol^39^. Considering the location of the NLRP3 receptor, sAC may take advantage of its location in interacting with the NLRP3 inflammasome. Thus, the present study indicates that compartmentalized cAMP, rather than total cAMP in the cytoplasm, may serve as a crucial factor in acetate-mediated attenuation of the NLRP3 inflammasome, supplementing the current theory of Ca^2+^/cAMP-mediated NLRP3 inflammasome attenuation.

Post translational modification has emerged as an endogenous regulatory mechanism of the inflammasome^42^, and several investigations have reported that ubiquitination acts as a negative regulator of NLRP3 inflammasome activation in a cAMP-PKA-dependent manner^6,41^. In this study, a similar pathway of PKA was also proven to be essential in acetate-induced ubiquitination of the NLRP3 inflammasome, and acetate subsequently induced inflammasome degradation through autophagy, rather than the proteasome. Thus, the present study suggests that acetate may bind to GPR43 and signal through sAC-PKA to attenuate the inflammasome.

Our data also showed that acetate plays a protective role in vivo. A previous study found that suppressing the NLRP3 inflammasome by blocking PKM2-dependent glycolysis protects mice from endotoxemia and polymicrobial sepsis^43^. This protective effect of acetate may be consistent. On the one hand, NLRP3 is reported to be involved in endotoxemia^44^ and acetate attenuates NLRP activation. On the other hand, our data also suggest that acetate somewhat attenuates LPS-induced NF-κB signaling activation (Fig. S2). Thus, by showing the protective effects of acetate in LPS-induced endotoxemia and the NLRP3 inflammasome-dependent peritonitis model, the present study may serve as the basis for more extensive research on acetate in sepsis and other inflammatory diseases.

In summary, in the present study, we demonstrate that acetate and GPR43 signaling attenuate NLRP3 inflammasome activation, and we report a novel mechanism of inflammasome regulation by metabolites. Additionally, our results reveal a critical role for sAC in NLRP3 inflammasome activation, which supplements the present mechanism of NLRP3 inflammasome regulation. The regulatory effect of acetate on inflammation provides an example of the mechanisms by which metabolites regulate immune responses and may offer a new therapeutic strategy for the clinical management of inflammatory diseases.

## Supplementary information

Supplementary Figure 1.

Supplementary Figure 2.

Supplementary Figure 3.

Supplementary Figure 4.

Supplementary Video 1.

Supplementary Video 2.

Supplementary Video 3.

Supplementary Video 4.
