# Publisher Correction: Acetate attenuates inflammasome activation through GPR43-mediated Ca^2+^-dependent NLRP3 ubiquitination

**DOI:** 10.1038/s12276-019-0296-1

**Published:** 2019-08-14

**Authors:** Mengda Xu, Zhengyu Jiang, Changli Wang, Na Li, Lulong Bo, Yanping Zha, Jinjun Bian, Yan Zhang, Xiaoming Deng

**Affiliations:** 1 Faculty of Anesthesiology, Changhai Hospital, Second Military Medical University, 200433 Shanghai, China; 2 Department of Anesthesiology, Wuhan General Hospital, PLA, 430070 Wuhan, Hubei Province China

**Keywords:** Acute inflammation, Phosphoinositol signalling, Inflammasome

**Correction to: Experimental & Molecular Medicine**

10.1038/s12276-019-0276-5, Published online 23 July 2018

This Article contains an error in the order of the figures. All figures except Fig. 4 were in wrong position. The figure legends are correct.

This has been corrected in both the PDF and HTML versions of the article.

