# Supplementary figures and images for: Acetate attenuates inflammasome activation through GPR43-mediated Ca2+-dependent NLRP3 ubiquitination
Source: Exp Mol Med. 2019 Jul 23;51(7):83. doi: 10.1038/s12276-019-0276-5 (PMC6802670; doi:10.1038/s12276-019-0276-5)

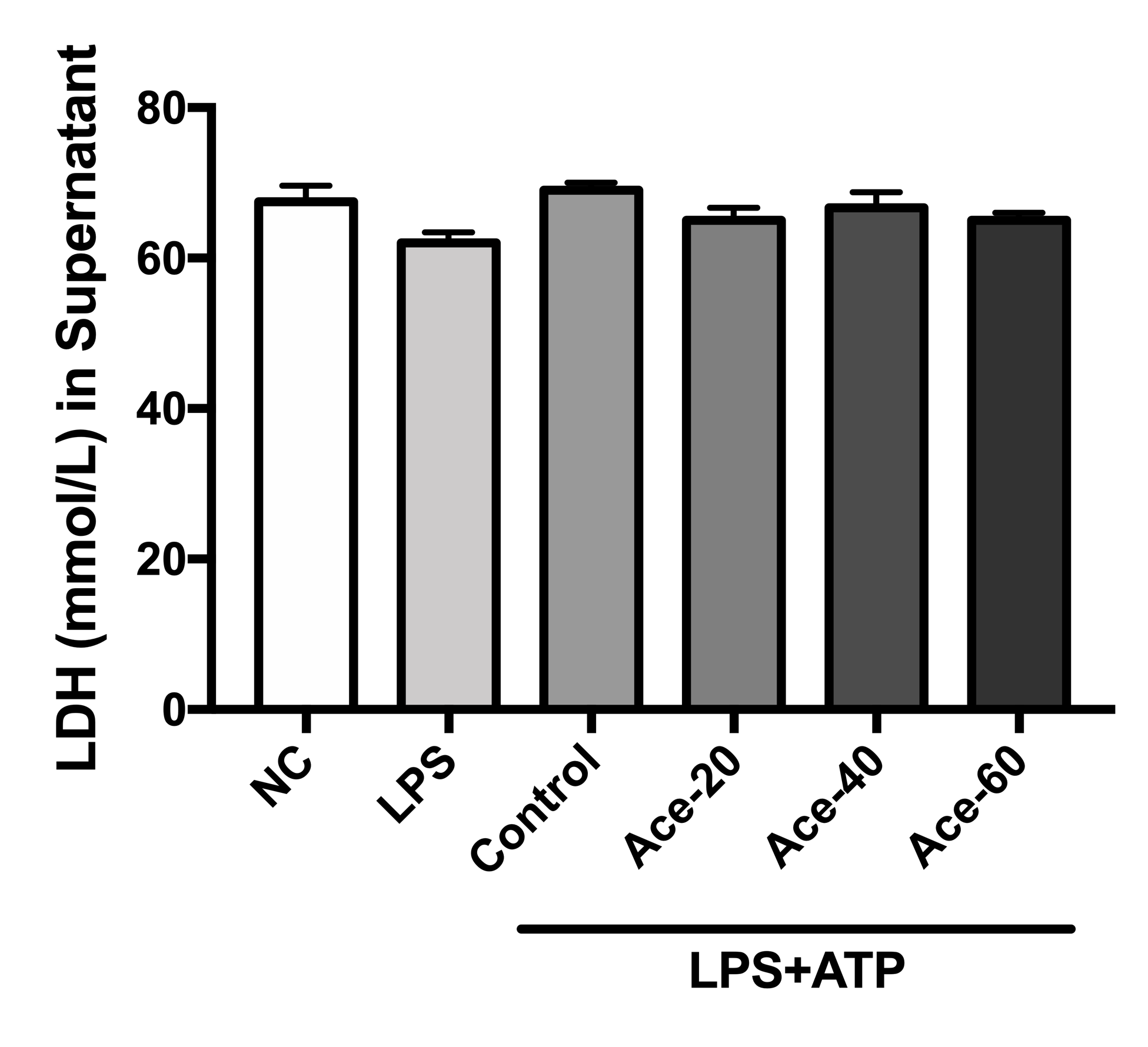

Supplement: Supplementary file 1 — Supplementary Figure 1. [file 12276_2019_276_MOESM1_ESM.tiff]

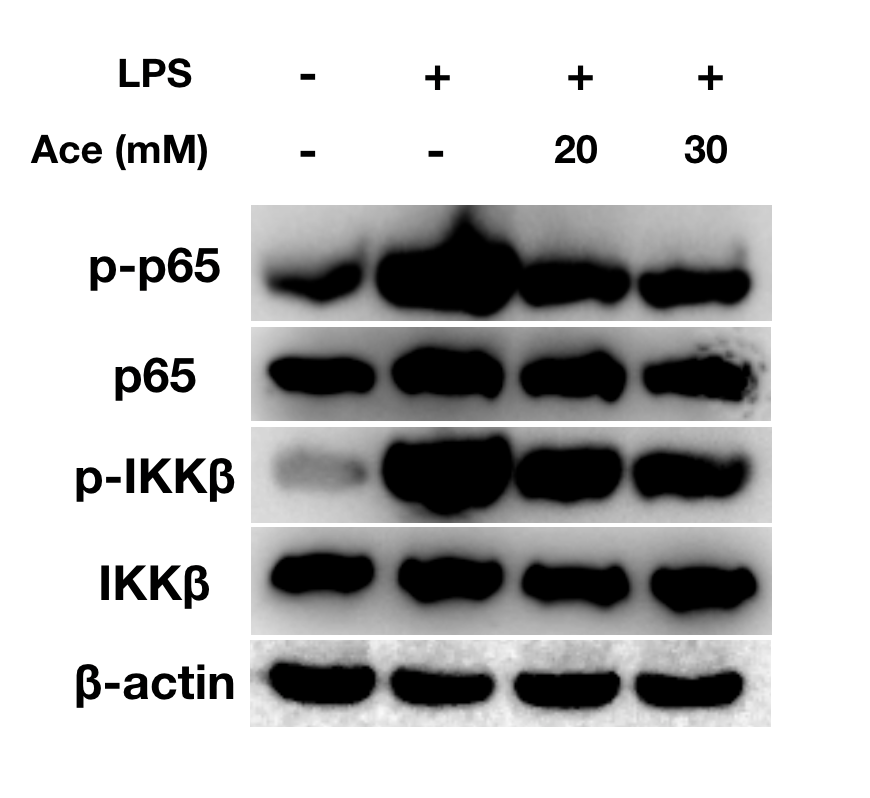

Supplement: Supplementary file 2 — Supplementary Figure 2. [file 12276_2019_276_MOESM2_ESM.png]

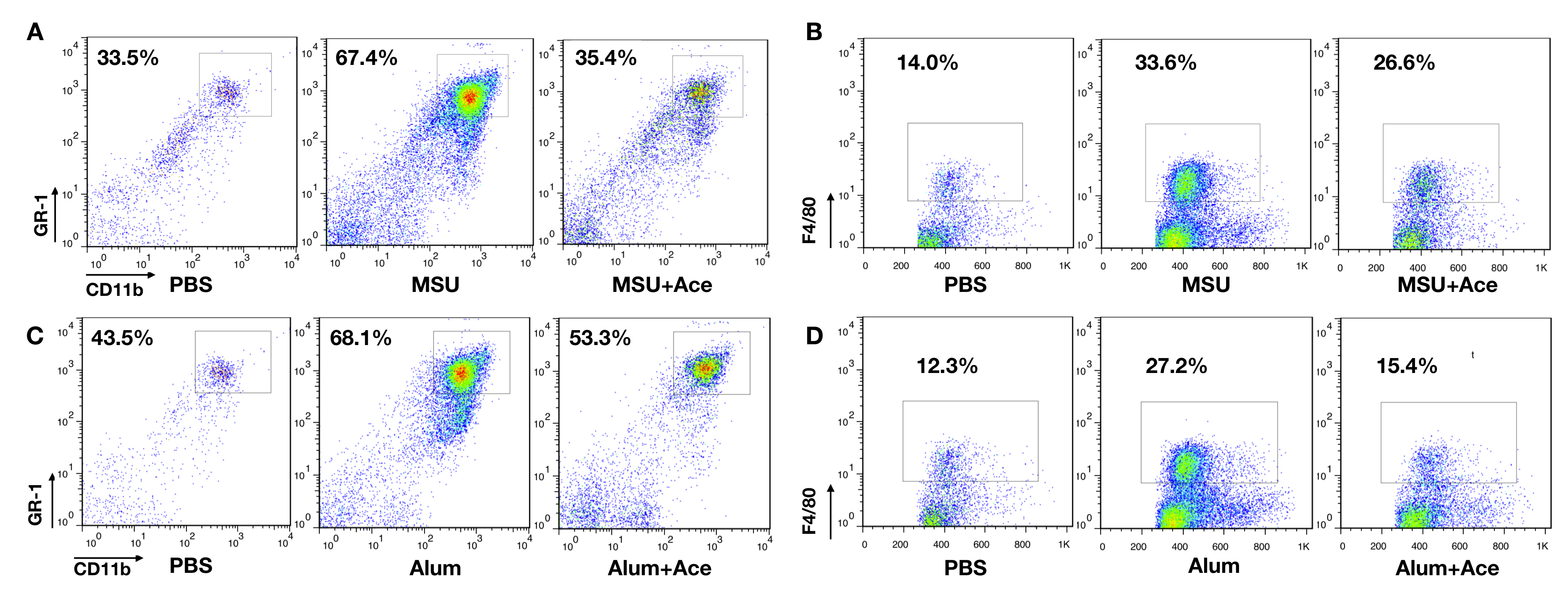

Supplement: Supplementary file 3 — Supplementary Figure 3. [file 12276_2019_276_MOESM3_ESM.tiff]

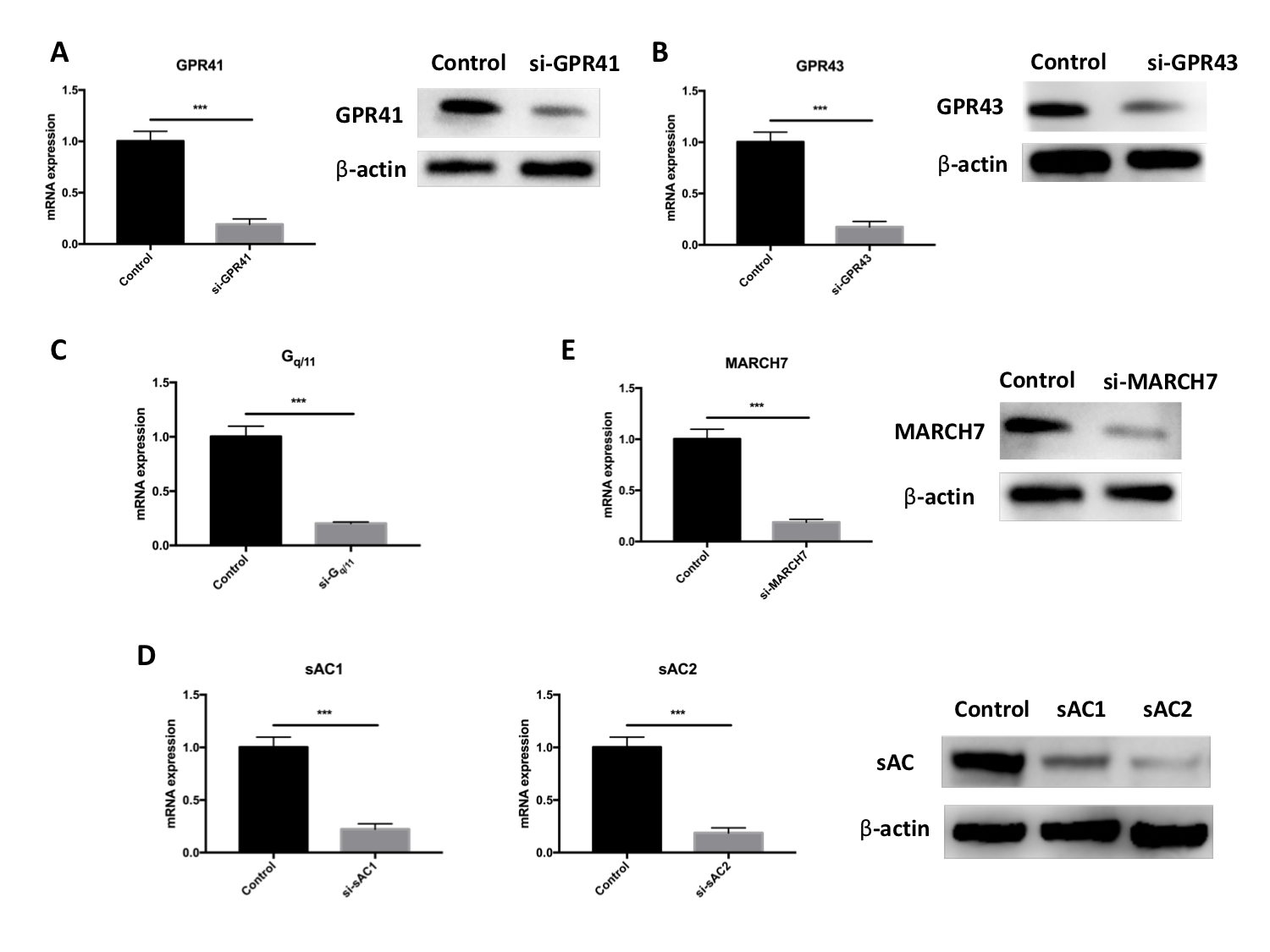

Supplement: Supplementary file 4 — Supplementary Figure 4. [file 12276_2019_276_MOESM4_ESM.tiff]
